# Supplementary material for: JWST interferometric imaging reveals the dusty torus obscuring the supermassive black hole of Circinus galaxy
Source: Nat Commun. 2026 Jan 13;17:42. doi: 10.1038/s41467-025-66010-5 (PMC12800092; doi:10.1038/s41467-025-66010-5)
Supplement: Supplementary file 1 — Supplementary Information [file 41467_2025_66010_MOESM1_ESM.pdf]

JWST interferometric imaging reveals the dusty torus obscuring the supermassive black hole of the Circinus galaxy

ENRIQUE LOPEZ-RODRIGUEZ, JOEL SANCHEZ-BERMUDEZ, OMAIRA GONZ ALEZ-MARTIN, ROBERT NIKUTTA, RYAN M. LAU, DEEPASHRI THATTE, ISMAEL GARCIA-BERNETE, JULIEN H. GIRARD, AND MATTHEW J. HANKINS.

Supplementary Information File

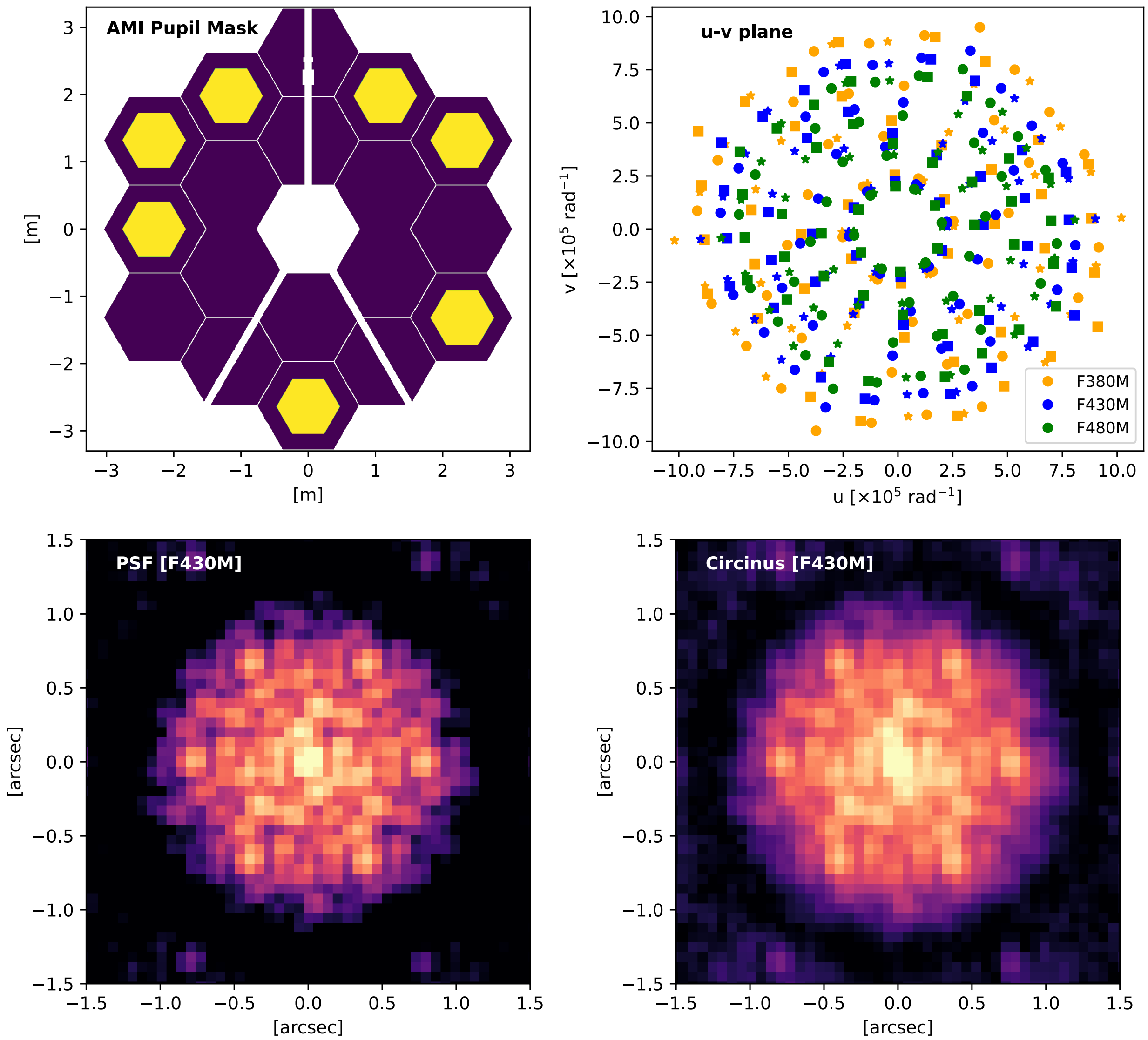

Supplementary Figure 1. Example of AMI/JWST observations. The 7-hole non-redundant NIRISS AMI pupil mask (yellow hexagons) over the JWST primary mirror (top left). The u-v coverage (top right) of the Circinus observations in the F380M (orange), F430M (blue), and F480M (green) filters. The u-v coverage for the first (circles) and second (squares) epochs is shown. The central 3″ × 3″ (45 × 45 px^2) interferogram image on the 80 × 80 px^2 SUB80 array with the F430M filter of the standard star HD119164 (bottom left) and Circinus (bottom right). The FOV of the Circinus observations is 90 × 90 pc^2. Both interferogram images are normalized to the peak. Compared with the standard star, the Circinus observations show an elongated feature within the central 0.5″ and a large-scale extended emission.

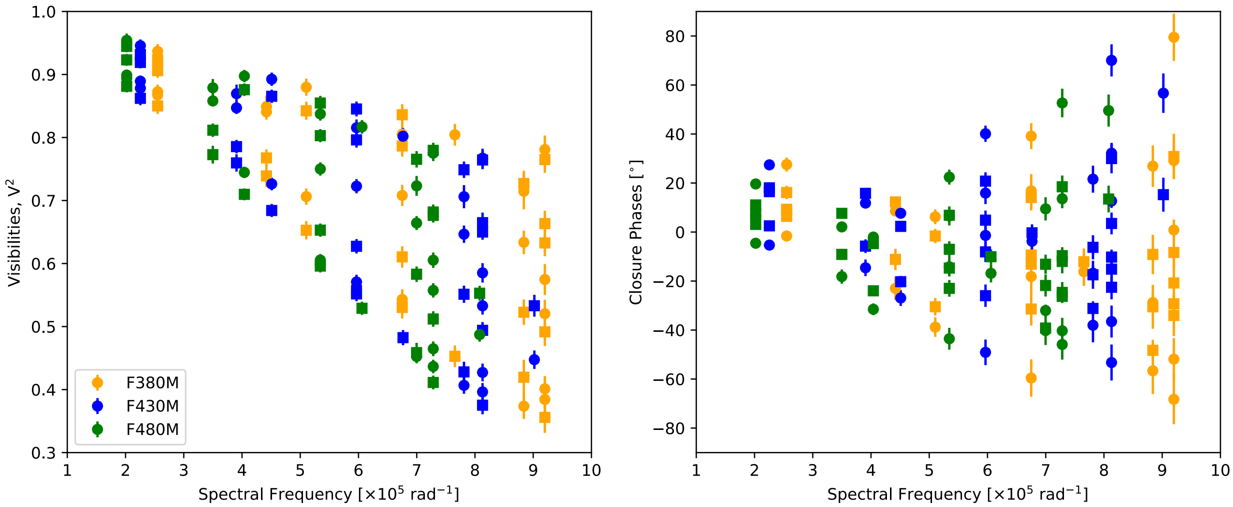

Supplementary Figure 2. Interferometric observables of Circinus. The calibrated visibilities (left) and closure phases (right) for the Circinus observations in the F380M (orange), F430M (blue), and F480M (green) filters for the first (circles) and second (squares) epochs. Error bars are the 1σ standard deviation of the measurement.

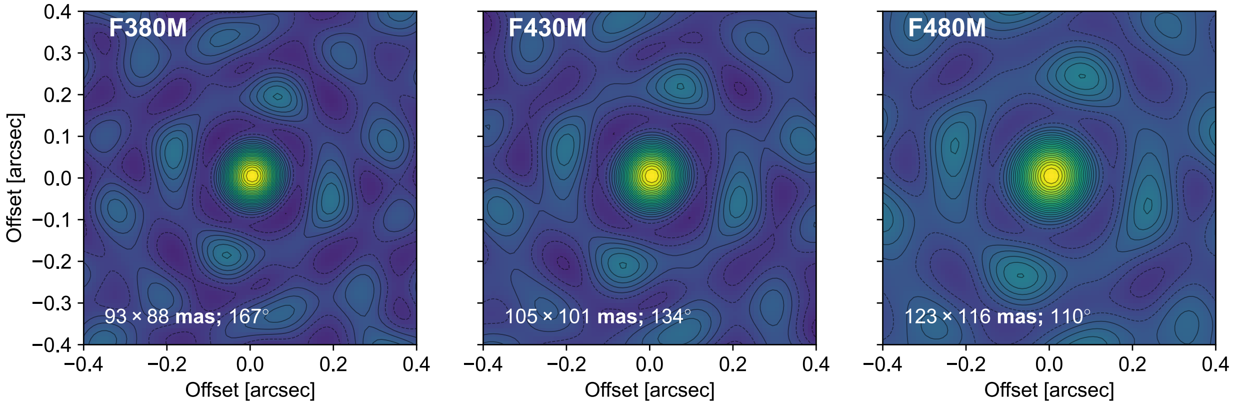

Supplementary Figure 3. Dirty Beams. The dirty beams of the F380M (left), F430M (middle), and F480M (right) filters. Dirty beams of the combined epochs. The FWHMs and PA of the beams are shown at the bottom left of each panel. Contours start at $-0.2 \times I_{peak}$ and increase in steps of 0.05, where I_{peak} is the peak of the beam scaled to unity.

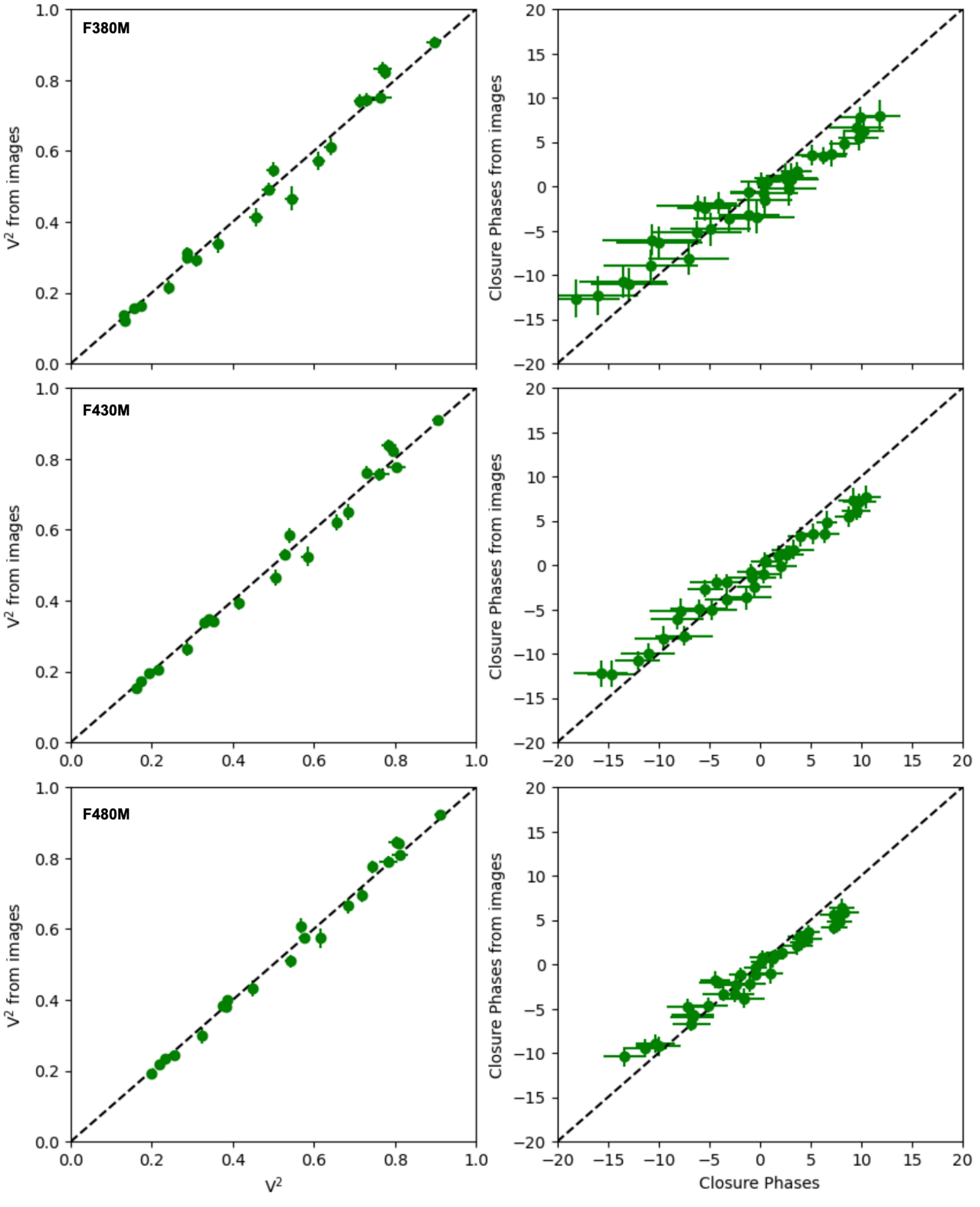

Supplementary Figure 4. Comparison between observables and synthetic interferometric observables. The amplitudes (left) and closure phases (right) for the F380M (top), F430M (middle), and F480M (bottom) filters using the bootstrapping analysis described in the Methods section 'Image reconstruction'. Error bars are the 1σ standard deviation of the measurement.

Table 1. Photometric measurements.

Filter	Angular Aperture	Physical Aperture	Flux Density	Contribution ext. emission	Flux density 'North arc'	Contribution 'North arc'
	[mas]	[pc]	[mJy]	[%]	[mJy]	[%]
(a)	(b)	(c)	(d)	(e)	(f)	(g)
F380M	93	1.9	250 ± 10	-	-	-
	123	2.5	360 ± 20	-	-	-
	$81 \times 162, -66°$	1.7×3.3	1050^{+60}_{-70}	13^{+5}_{-6}	14.1 ± 0.7	~ 1
	640	12.3	1180 ± 59	-	-	-
F430M	105	2.1	400 ± 20	-	-	-
	123	2.5	510 ± 30	-	-	-
	$84 \times 177, -68°$	1.7×3.6	1670^{+90}_{-80}	13^{+4}_{-5}	18.9 ± 0.9	~ 1
	640	12.3	1870 ± 94	-	-	-
F480M	123	2.5	940 ± 80	-	-	-
	$90 \times 183, -71°$	1.8×3.7	3030^{+150}_{-150}	11^{+4}_{-4}	21.0 ± 1.7	< 1
	640	12.3	3360 ± 269	-	-	-

NOTE—Columns, from left-hand to right-hand: (a) AMI/JWST filter, (b) angular circular aperture in units of mas and the x and y axes and PA of the 2D Gaussian profile, (c) physical circular aperture and the x and y axes and PA of the 2D Gaussian profile, (d) flux density in units of mJy within the circular aperture and integrated under the full 2D Gaussian profile, (e) fractional contribution of all the extended emission after removal of the central 2D Gaussian profile, (f) flux density in units of mJy of the 'North arc' feature with a radius of 76 mas (1.5 pc), and (g) fractional contribution of the 'North arc' feature within the central 10×10 pc^2

Supplementary Table 1. Photometric measurements.

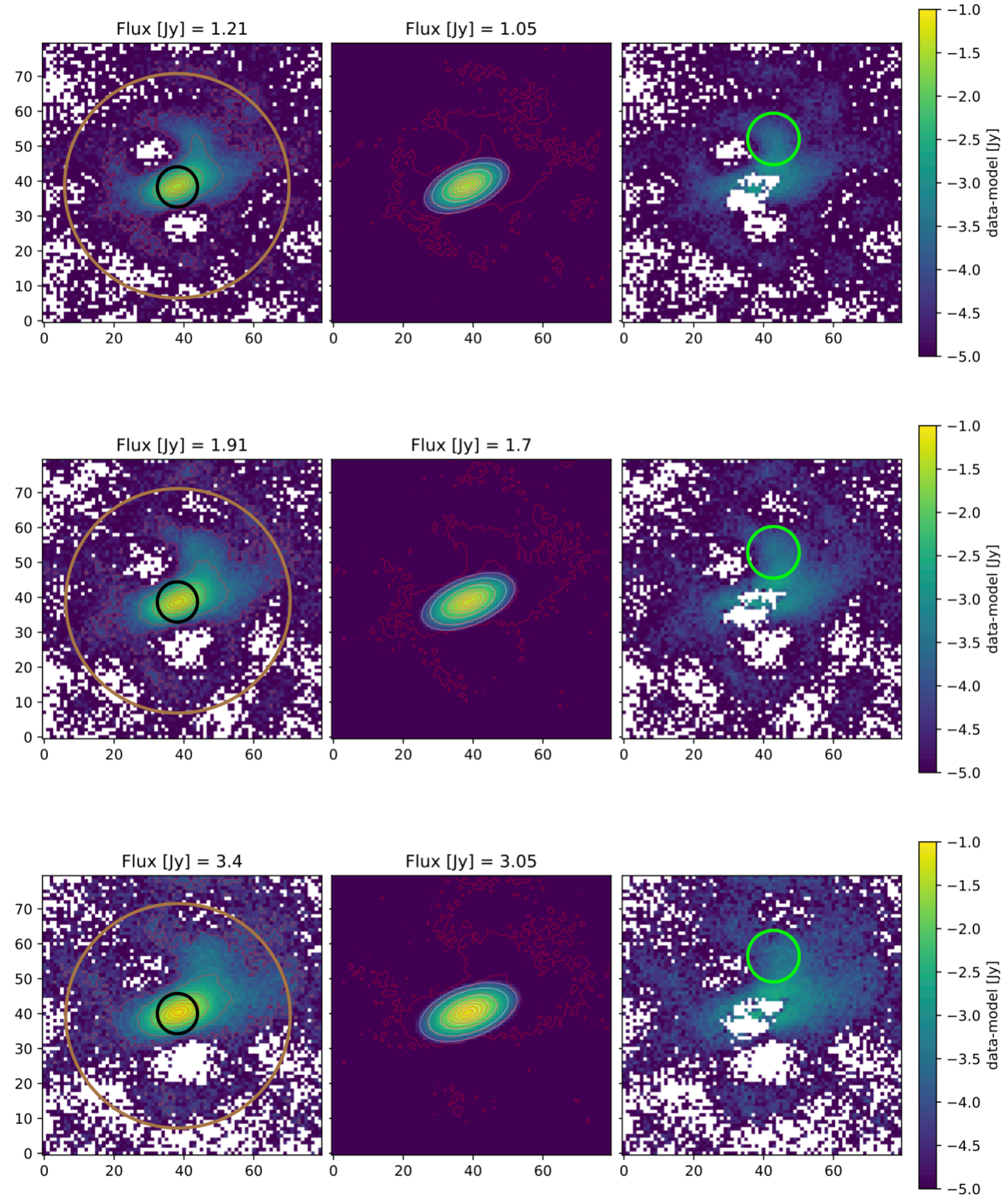

Supplementary Figure 5. The results of the 2D Gaussian fitting to the extended emission in each of the AMI/JWST observations. The AMI/JWST observations (first column), best-fit 2D Gaussian model (middle column), and residuals (third column) for the F380M (first row), F430M (middle row), and F480M (bottom row) filters. The red contours show the flux density of the observations, and the white contours show the fluxes of the 2D Gaussian profiles. All contours have the same fractional levels from the peak pixel at [10−3, 10−2, 10−1, 0.3, 0.5, 0.7, 0.9]. The axes are in pixels with a pixel scale of 10 mas. The total flux densities in log-scale, within the FOV of 14 × 14 pc^2 are shown. The apertures used in Supplementary Table 1 of 123 mas (black circle), 640 mas (brown circle) centered on the AGN, and the 152 mas (green circle) covering the 'North Arc' are shown.

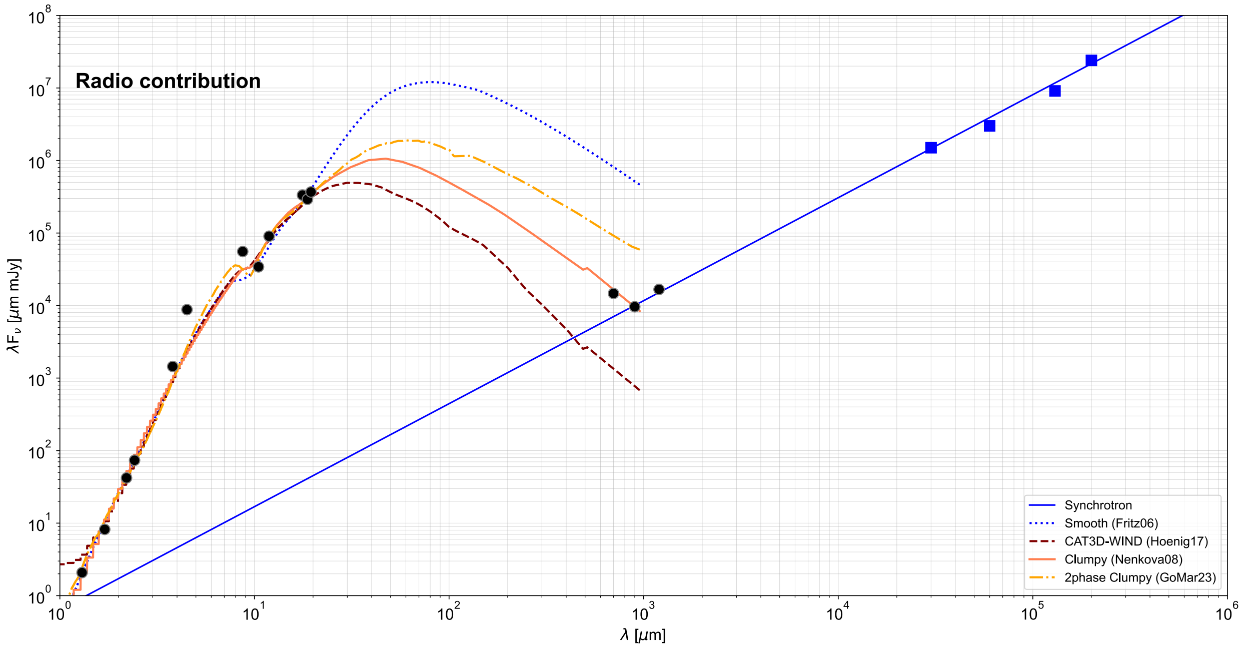

Supplementary Figure 6. Radio contribution and torus models. The Circinus SED (black circles) and the radio observations from ATCA (blue squares). The synchrotron emission passing through the ATCA data and the 1200 μm photometric points are shown (blue solid line).

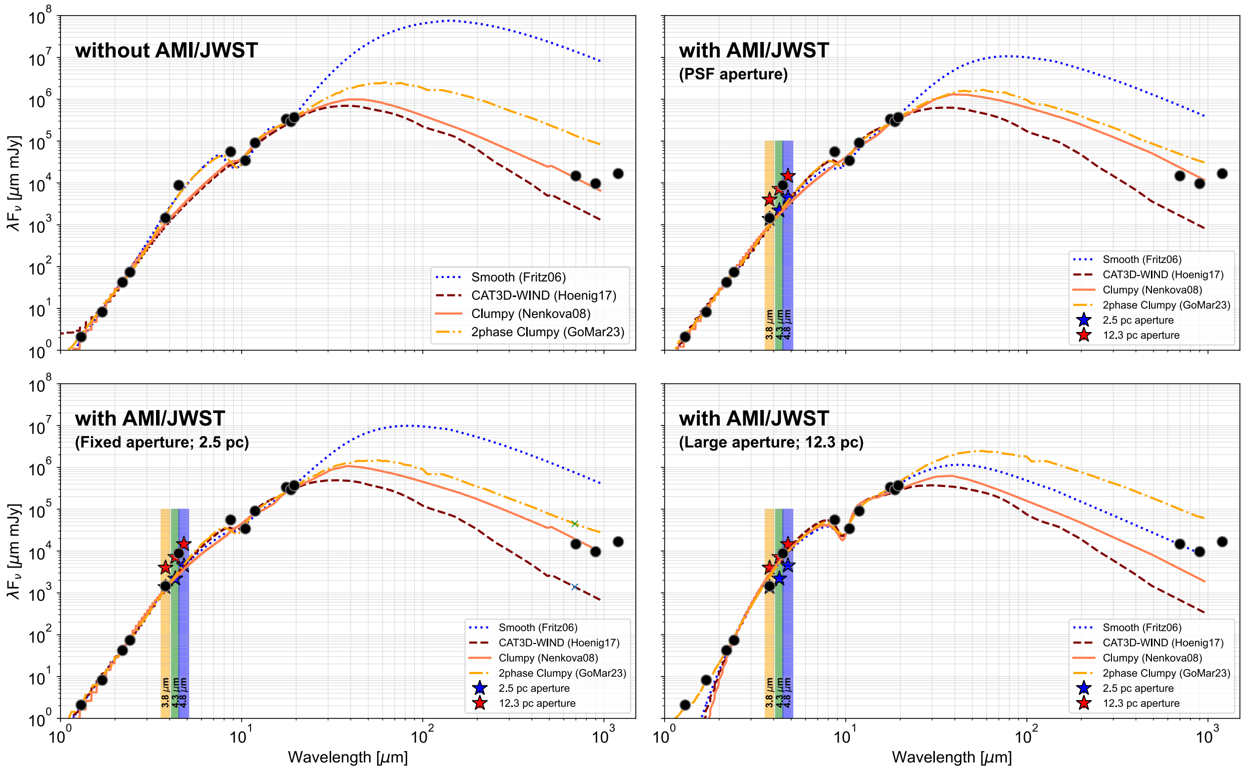

Supplementary Figure 7. The 1–1000 μm SED of Circinus with best-fit torus models. The SED without AMI/JWST data (top-left) and with the AMI/JWST photometric measurements fitting the PSF aperture (top-right), a fixed aperture of 1.5 pc (bottom left), a large aperture of 12.3 pc (bottom right) are shown. In each panel, we show the archival data (black circles), AMI/JWST photometry (stars), and the best-fit torus models as shown in the legend.

					CLUMPY (Nenkova08)						
	i	N_0	σ	Y	q	τ_V	E(B-V)			χ^2_{ALL}	χ^2_{IR}
without AMI/JWST	90°	4^{+1}_{-1}	$< 70^\circ$	< 100	$1.6^{+0.3}_{-0.3}$	24^{+8}_{-5}	< 0.11			1.24	1.23
with AMI/JWST (psf)	90°	4^{+3}_{-1}	$< 70^\circ$	< 95	$1.7^{+0.4}_{-0.3}$	30^{+4}_{-3}	< 0.19			3.64	3.21
with AMI/JWST (1.5pc)	90°	4^{+1}_{-1}	$< 70^\circ$	< 100	$1.6^{+0.3}_{-0.3}$	20^{+3}_{-2}	< 0.12			2.95	2.93
with AMI/JWST (8pc)	90°	< 2	$60^{+10\circ}_{-45}$	5^{+2}_{-1}	< 2.5	59^{+12}_{-23}	$15.1^{+0.6}_{-0.7}$			6.54	3.66
					Smooth (Fritz06)						
	i	σ	γ	β	Y	τ_V	E(B-V)			χ^2_{ALL}	χ^2_{IR}
without AMI/JWST	90°	33^{+5}_{-5}	< 0.01	< -1	56^{+14}_{-3}	< 10	< 0.45			9×10^5	0.75
with AMI/JWST (psf)	90°	23^{+2}_{-3}	< 0.01	< -1	47^{+4}_{-9}	< 10	< 0.13			2×10^3	2.90
with AMI/JWST (2.5pc)	90°	30^{+3}_{-9}	< 0.01	< -1	55^{+23}_{-3}	< 10	< 0.22			2×10^3	2.28
with AMI/JWST (12.3pc)	90°	23^{+9}_{-3}	$2.0^{+0.3}_{-0.7}$	< -1	> 10	$9.9^{+0.1}_{-1.5}$	$7.7^{+0.9}_{-0.6}$			3.02	3.01
					2-phase clumpy (GoMar23)						
	i	σ	p	q	Y	τ_V	$P_{\rm size,max}$	E(B-V)		χ^2_{ALL}	χ^2_{IR}
without AMI/JWST	90°	$< 11^\circ$	> 1.3	< 1.5	28^{+2}_{-7}	6^{+1}_{-1}	$0.06^{+0.01}_{-0.01}$	< 0.28		72.14	0.61
with AMI/JWST (psf)	90°	$< 80^\circ$	> 1.5	> 1.0	24^{+4}_{-2}	5^{+1}_{-1}	$0.10^{+0.05}_{-0.01}$	$1.1^{+0.2}_{-0.1}$		8.70	2.67
with AMI/JWST (2.5pc)	90°	$< 80^\circ$	> 1.3	< 1.5	25^{+4}_{-3}	5^{+1}_{-1}	$0.08^{+0.01}_{-0.01}$	$0.69^{+0.24}_{-0.20}$		6.54	2.12
with AMI/JWST (12.3pc)	90°	$< 80^\circ$	> 1.4	> 1.4	29^{+1}_{-2}	6^{+1}_{-1}	$0.05^{+0.01}_{-0.01}$	< 0.07		37.99	1.40
					CAT3D-WIND (Hoenig17)						
	i	N_0	a	θ	σ_θ	a_w	h	fwd	E(B-V)	χ^2_{ALL}	χ^2_{IR}
without AMI/JWST	90°	$5.3^{+0.8}_{-0.3}$	$-1.9^{+0.3}_{-0.2}$	$45^{+1\circ}_{-7}$	$14^{+2\circ}_{-2}$	< -2.5	> 0.1	$0.6^{+0.1}_{-0.1}$	$2.5^{+0.2}_{-0.4}$	2.44	1.79
with AMI/JWST (psf)	90°	> 5	$-1.6^{+0.1}_{-0.2}$	$< 45^\circ$	$< 15^\circ$	< -2.5	> 0.1	$0.5^{+0.6}_{-0.6}$	$2.2^{+0.2}_{-0.2}$	3.69	2.91
with AMI/JWST (2.5pc)	90°	> 5	$-1.8^{+0.1}_{-0.2}$	$34^{+11\circ}_{-4}$	$< 15^\circ$	< -2.5	> 0.1	$0.6^{+0.6}_{-0.1}$	$2.3^{+0.3}_{-0.2}$	3.38	2.53
with AMI/JWST (12.3pc)	90°	7^{+3}_{-2}	$-2.6^{+0.1}_{-0.1}$	$< 30^\circ$	$< 15^\circ$	< -2.5	> 0.1	< 0.75	$10.8^{+0.6}_{-1.0}$	4.04	3.13

Supplementary Table 2. Best-fit torus model parameters. The inclination is fixed to 90◦ with the rest of the parameters to be set free. X^2_{ALL} is the χ^2 for the 1−1000 μm SED; χ^2_{IR} is the χ^2 for the 1 − 20 μm SED.

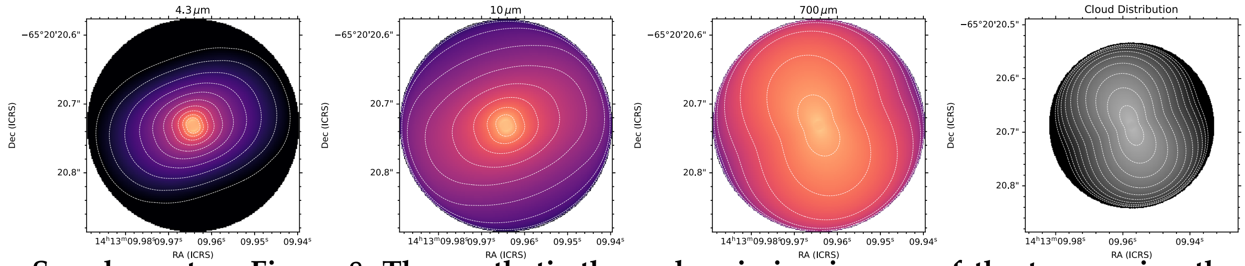

Supplementary Figure 8. The synthetic thermal emission images of the torus using the best-fit CLUMPY torus models. From left to right, the 4.3 μm, 10 μm, 700 μm thermal emission distributions, and the cloud distribution of the torus. The images are shown at the native resolution of the model. Figure 3 shows the same images after smoothing using a 2D Gaussian profile equal to the beam of the observations at each wavelength.
